# Use of power-law analysis to predict abuse or diversion of prescribed medications: proof-of-concept mathematical exploration

**DOI:** 10.1186/s13104-018-3632-y

**Published:** 2018-07-31

**Authors:** Kathleen A. Fairman, Alyssa M. Peckham, Michael L. Rucker, Jonah H. Rucker, David A. Sclar

**Affiliations:** 1 College of Pharmacy-Glendale, Midwestern University, 19555 N 59th Ave, Glendale, AZ 85308 USA; 2 Northeastern University School of Pharmacy and Massachusetts General Hospital, Boston, USA; 3 Wood Environment & Infrastructure Solutions, Phoenix, USA; 4 Kathleen Fairman LTD, Phoenix, USA

**Keywords:** Gabapentin, Alprazolam, Opioids, Abuse, Diversion, Power-law analysis, Lorenz-curve analysis

## Abstract

**Objective:**

To conduct a proof-of-concept study comparing Lorenz-curve analysis (LCA) with power-law (exponential function) analysis (PLA), by applying segmented regression modeling to 1-year prescription claims data for three medications—alprazolam, opioids, and gabapentin—to predict abuse and/or diversion using power-law zone (PLZ) classification.

**Results:**

In 1-year baseline observation, patients classified into the top PLZ groups (PLGs) were demographically and diagnostically similar to those in Lorenz-1 (top 1% of utilizers) and Lorenz-25 (top 25%). For prediction of follow-up (6-month post-baseline) Lorenz-1 use of alprazolam and opioids (i.e., potential abuse/diversion), PLA had somewhat lower sensitivity compared with LCA (83.5–95.4% vs. 99.5–99.9%, respectively) but better specificity (98.2–98.8% vs. 75.5%) and much better positive predictive value (PPV; 34.5–45.3% vs. 4.0–4.6%). Of top-PLG alprazolam- and opioid-treated patients, respectively, 20.7 and 9.9% developed incident (new) Lorenz-1 in followup, compared with < 3% of Lorenz-25 patients. For gabapentin, neither PLA nor LCA predicted incident Lorenz-1 (PPV = 0.0–1.4%). For all three medications, PLA sensitivity for follow-up hospitalization was < 5%, but specificity was better for PLA (97.3–99.2%) than for LCA (74.3–75.4%). PLA better identified patients at risk of future controlled substance abuse/diversion than did LCA, but the technique needs refinement before widespread use.

**Electronic supplementary material:**

The online version of this article (10.1186/s13104-018-3632-y) contains supplementary material, which is available to authorized users.

## Introduction

For the United States (US) health care system, identifying and intervening on patients who are abusing or diverting controlled substances is a top priority because of high cost, morbidity, and mortality associated with fraud, waste, or abuse (FWA) [1, 2]. Numerous methods for identifying at-risk patients have been proposed [3–5]. Limiting their utility, most methods require integrated medical/pharmacy claims datasets, laboratory data, or complicated algorithms that may exceed the programming resources available in many health care organizations [3–5].

Various simpler analyses based solely on pharmacy claims data have been suggested [3, 6]. One common technique is Lorenz-curve analysis (LCA), which assesses the percentage of total medication supply dispensed to top utilizers, with Lorenz-1 indicating supply dispensed to the top 1% [6–8]. A Lorenz-1 of 15% or more indicates an abusable medication [8].

Although the Lorenz-1 metric is intuitively appealing and easily calculated, it indicates FWA that is already ongoing, perhaps at a dangerous level. One study of Lorenz-1 utilizers found dispensed dosages averaging 11,274 mg/day for gabapentin and 180 morphine-milligram equivalents (MME)/day for opioids [9], with both > 3 times the labeled/recommended dosage for any indication [10]. A follow-up study found that concomitant high-dosage consumption/diversion of gabapentin and opioids approximately doubled the risk of inpatient hospitalization (IPH) and quadrupled the risk of respiratory depression-related emergency or IPH care [11]. Thus, although important, Lorenz-1 may indicate damage that has already taken place. Ideally, health care systems would be able to predict medication FWA before it begins, acting on warning signs of future events.

One technique proposed to make predictions of this type, adapted from geophysical sciences, is power-law analysis (PLA), also known as fractal-scaling analysis [12], which was originally developed to predict catastrophic hurricanes, earthquakes, and other geophysical hazards [13]. In PLA, logarithmically transformed 2-dimensional plots of cumulative frequency against event magnitude are segmented into zones, each characterized by a linear equation (power law). “Transitions” (i.e., points at which the power law changes) indicate fundamental transformations in the nature of the events [12]. For example, a transition in the power law for hurricane wind speed magnitudes, identified by an early pioneer of the technique, indicated eyewall formation [12].

The present study was a proof-of-concept comparison of LCA with PLA, applied to prescription claims data for 3 medications: alprazolam, the benzodiazepine with the greatest abuse liability; [14, 15] opioids, which are a top public health concern because of an epidemic of misuse and overdose deaths; [1, 16] and gabapentin, a noncontrolled substance recently identified as a medication of abuse [10, 11].

## Main text

### Methods

#### Data source and sample

Study data were derived from the Truven Health Market Scan^®^ Commercial Claims and Encounters Database for calendar years 2013–2015. The database, which comprises pharmacy claims, medical claims, and eligibility (enrollment) data for up to 50 million commercially insured enrollees annually, is commonly used in studies of US health care [17].

The study sample included enrollees aged 16–64 years with ≥ 2 claims for alprazolam, gabapentin, and/or any opioid during a 365-day period that began with the first observed pharmacy claim (baseline treatment year). To ensure accurate dosage calculations, patients using patches or fentanyl, or with missing or invalid dosages on any claims, were excluded. Patients using multiple study medications were *not* excluded; instead, each medication had its own baseline year, and concomitant uses of other study medications were measured.

#### Cohort group definitions

Based on utilization in the baseline treatment year, patients were categorized into groups using both PLA and LCA, including Lorenz-1 (top 1% of users) and Lorenz-25 (top 25%). These classifications were performed using SPSS v24.0 (IBM SPSS, Armonk, NY).

Specifically, for each patient and medication, total supply dispensed during the baseline year was summed as total mg for alprazolam and gabapentin and total MME for opioids, across all claims and strengths. For the LCA, again for each medication, patients were ranked according to total dispensed supply; and Lorenz-1 and quartile groups, including Lorenz-25, were identified.

The PLA also used rankings based on summed supply data, but patients were grouped differently: into 5-‰ bands from the 5th to 95th percentile, and into more granular (smaller) bands at the lowest and highest ends of the percentile distribution. Specifically, at the lowest end, patients were grouped as < 1st percentile and 1st  < 5th percentile. At the highest end, patients were grouped into single-percentage increments (e.g., 96th, 97th, etc.) to 99th, then into bands representing one-tenth of 1 percent increments (e.g., 99.1, 99.2%, etc.) up to the 99.9th percentile. This approach was taken because PLA predicts rare events, making a granular analysis of top utilizers necessary to determine the power law for each linear segment.

Summed data were then loaded into Excel (Microsoft, Redmond, WA). Power-law calculations, including identification of transition points and power-law zones (PLZs), were performed in Excel using a method similar to that of geophysical sciences for event magnitude and frequency [12, 13]. Specifically, for each utilization band, ranked from highest to lowest magnitude (i.e., dosage), medication supply/patient was calculated (summed supply ÷ summed patient count). Then, again for each band, *patient frequencies* (counts) were accumulated, whereas *dosage magnitudes* (supply/patient) were *not* accumulated. Both cumulative frequencies and magnitudes for each band were then log-transformed (log_10_; Additional file 1: Appendix S1). The resulting values were plotted, with the x-axis representing log_10_-magnitude, and the y-axis representing log_10_-cumulative frequency. Using a segmented-regression approach [18], PLZs were identified by visual inspection coupled with model fitting using linear regression.

#### Cohort group analyses

Two types of analyses were performed in SPSS, each including comparisons of groups based on LCA quartiles and on PLZs. The first assessed patient characteristics in the baseline treatment year. The second, a criterion validity assessment, was limited to subgroups of patients continuously enrolled through the 6 months *after* the baseline year (follow-up). Follow-up outcomes included Lorenz-1 utilization (i.e., potential FWA), IPH, and dosages/day standardized as Z-scores (distance from the mean measured in standard deviation units). Top-PLZ groups (PLGs) and top-LCA quartiles at *baseline* were defined as “at risk”; and rates of sensitivity, specificity, positive predictive value (PPV), and negative predictive value were calculated based on *follow*-*up* outcomes.

### Results

For alprazolam, 4 separate power-law zones (linear segments) were identified, with R^2^ ranging from 0.990 to 0.999. For gabapentin and opioids, 3 zones were identified, with R^2^ = 0.961–0.998 (Additional file 1: Appendix S2, Additional file 1: Appendix S3).

#### Patient characteristics, baseline treatment year

For alprazolam and opioids, top PLGs were larger and used less medication compared with Lorenz-1 groups (Table 1; Additional file 1: Appendix S4). Threshold dosages for top-PLG and Lorenz-1, respectively, were 5.04 versus 7.40 mg/day alprazolam (i.e., 126 and 185% of maximum labeled/recommended dosage); and 130.2 versus 271.2 MME/day opioids (i.e., 260% vs. 542% of maximum labeled/recommended dosage). Of those in the top PLG, only 46.2% of alprazolam- and 36.9% of opioid-treated patients were Lorenz-1 in the baseline year. In all other respects, however, top PLG and Lorenz-1 patients were similar, demographically, diagnostically, and in proportions of claims exceeding recommended dosages. For example, comparing alprazolam-treated patients in PLG-4 and Lorenz-1, respectively, 63 and 64% were female; mean claims/month exceeding labeled/recommended dosage were 0.84 and 0.91; 21 and 20% were diagnosed with substance use disorder (SUD); and 59% in each group were diagnosed with anxiety. Similar patterns were observed in patients treated with opioids.

**Table 1:** Patient characteristics and utilization patterns by medication and group, baseline treatment year

	PLG1	PLG2	PLG3	PLG4	Lorenz-25	Lorenz-1
Alprazolam (n)	**283,970**	**187,266**	**57,854**	**11,662**	**137,047**	**5384**
% of sample	52.5	34.6	10.7	2.2	25.3	1.0
Threshold dosage/day^a^	N/A	0.33	1.69	5.04	0.90	7.40
Ratio threshold:maximum	N/A	0.08	0.42	1.26	0.23	1.85
Female (%)	73.0	66.9	63.6	63.2	64.1	63.9
Mean age	46	48	48	48	48	49
Mean claims > max dose/month^b^	0.13	0.22	0.33	0.84	0.33	0.91
Lorenz-1 (%)	0.0	0.0	0.0	46.2	3.9	100.0
Diagnoses and utilization^c^ %						
Anxiety	45.4	49.6	55.9	58.9	53.9	59.3
Cancer	6.1	6.3	5.7	5.7	5.9	5.8
Insomnia	12.2	14.5	14.4	15.5	14.6	15.3
Pain	55.8	61.4	66.8	68.2	65.2	67.8
SUD	8.1	12.9	18.5	20.6	16.9	19.6
IPH (% with ≥ 1)	7.1	9.5	11.8	11.6	11.0	10.1
Pain	2.2	3.3	4.5	4.6	4.1	4.1
SUD	1.3	2.4	3.6	4.0	3.2	3.3
Z drug hypnotic^d^	11.3	16.1	18.1	18.6	17.7	18.0
Gabapentin^c^	3.7	6.6	8.9	8.9	8.2	8.5
Opioid^c^	24.2	37.6	51.9	52.1	47.5	50.4
Gabapentin (n)	**208,848**	**106,213**	**2476**	N/A	**81,534**	**3266**
% of sample	65.8	33.4	0.8		25.7	1.0
Threshold dosage/day^a^	N/A	766.03	12,509.59		1034.25	10,356.16
Ratio threshold:maximum	N/A	0.21	3.47		0.29	2.88
Female (%)	64.6	61.7	59.1		61.2	60.1
Mean age	50	51	52		51	52
Mean claims > max dose/month^b^	0.00	0.06	0.80		0.10	0.77
Lorenz-1 (%)	0	0.7	100.0		4.0	100.0
Diagnoses and utilization^c^ %						
Anxiety	20.6	22.7	27.9		23.0	27.3
Cancer	8.1	8.5	8.6		8.5	8.4
Insomnia	12.2	13.8	17.6		14.0	17.2
Pain	84.4	86.4	90.0		86.7	89.7
SUD	12.9	15.8	21.2		16.4	20.5
IPH (% with ≥ 1)	14.1	16.2	17.1		16.4	16.7
Pain	7.9	9.6	10.9		9.8	10.6
SUD	2.9	3.4	5.0		3.5	4.7
Benzodiazepine^d^	21.3	26.6	27.8		26.9	27.2
Z drug hypnotic^d^	9.7	12.9	12.9		13.0	12.7
Opioid^c^	41.9	51.6	57.9		52.9	56.1
Opioids (n)	**2,172,054**	**217,918**	**67,514**	N/A	**615,003**	**24,884**
% of sample	88.4	8.9	2.7		25.0	1.0
Threshold dosage/day^a^	N/A	22.60	130.19		5.82	271.15
Ratio threshold:maximum	N/A	0.45	2.60		0.12	5.42
Female (%)	59.4	52.2	45.7		53.6	46.2
Mean age	45	48	47		48	49
Mean claims > max dose/month^b^	0.08	0.52	1.44		0.44	1.63
Lorenz-1 (%)	0	0	36.9		4.0	100.0
Diagnoses and utilization^c^ %						
Anxiety	14.5	24.5	25.7		22.3	26.2
Cancer	6.6	7.1	6.3		7.4	6.6
Insomnia	7.8	12.5	12.7		11.9	13.6
Pain	66.8	87.8	83.1		85.1	88.3
SUD	9.5	24.8	36.4		20.8	32.2
IPH (% with ≥ 1)	12.5	17.9	15.5		18.7	15.6
Pain	4.9	10.4	9.2		11.3	9.8
SUD	1.5	4.5	5.1		3.8	5.0
Benzodiazepine^d^	14.2	35.4	38.2		30.8	41.2
Z drug hypnotic^d^	6.4	14.8	14.4		13.2	15.2
Gabapentin^c^	4.9	16.9	16.5		14.1	17.0

*IPH* inpatient hospital stay, *Lorenz-1* top 1% of utilizers, *Lorenz-25* top quartile (25%) of utilizers, *mg* milligrams, *MME* morphine-milligram equivalents, *PLG* power-law group, *SUD* substance use disorder

^a^Medication supply was measured as milligrams for alprazolam (n = 540,752) and gabapentin (n = 317,537), and MMEs for opioids (n = 2,457,486). All medication claims were measured in the baseline treatment year (i.e., 12-month period beginning with the first observed medication claim of the type shown in the row label); sample is not limited to new utilizers. Threshold is the dosage that defines the category lower limit; for example, > 0.33 and ≤ 1.69 mg defined PLZ-2 alprazolam

^b^Total supply dispensed in each claim divided by days supply; rate was measured as total number of claims exceeding labeled/recommended dosage (4 mg/day alprazolam, 3600 mg/day gabapentin; 50 MME/day opioids), divided by 12

^c^Measured in the baseline treatment year. Diagnosis codes are shown in Additional file 1: Appendix S4

^d^Benzodiazepines measured: clonazepam, diazepam, lorazepam and, for users of gabapentin and opioids, alprazolam. Z-drugs measured: eszopiclone and zolpidem. Percentages of patients with ≥ 2 claims

In contrast, for those treated with gabapentin, the threshold dosage for the top PLG was *higher* than the Lorenz-1 threshold, at 12,510 and 10,356 mg/day, respectively; and 100% of PLG-3 patients were Lorenz-1 (Table 1). However, gabapentin PLG-3 and Lorenz-1 patients were similar in other respects.

For all 3 medications, Lorenz-25 patient groups were characterized by relatively low dosage thresholds (ranging from 12 to 29% of maximum; Table 1). Baseline SUD prevalence rates were generally lower for these groups than for the top PLGs and Lorenz-1 groups, but otherwise, the groups were demographically and diagnostically similar.

#### Criterion validity analyses

**Fig. 1:**
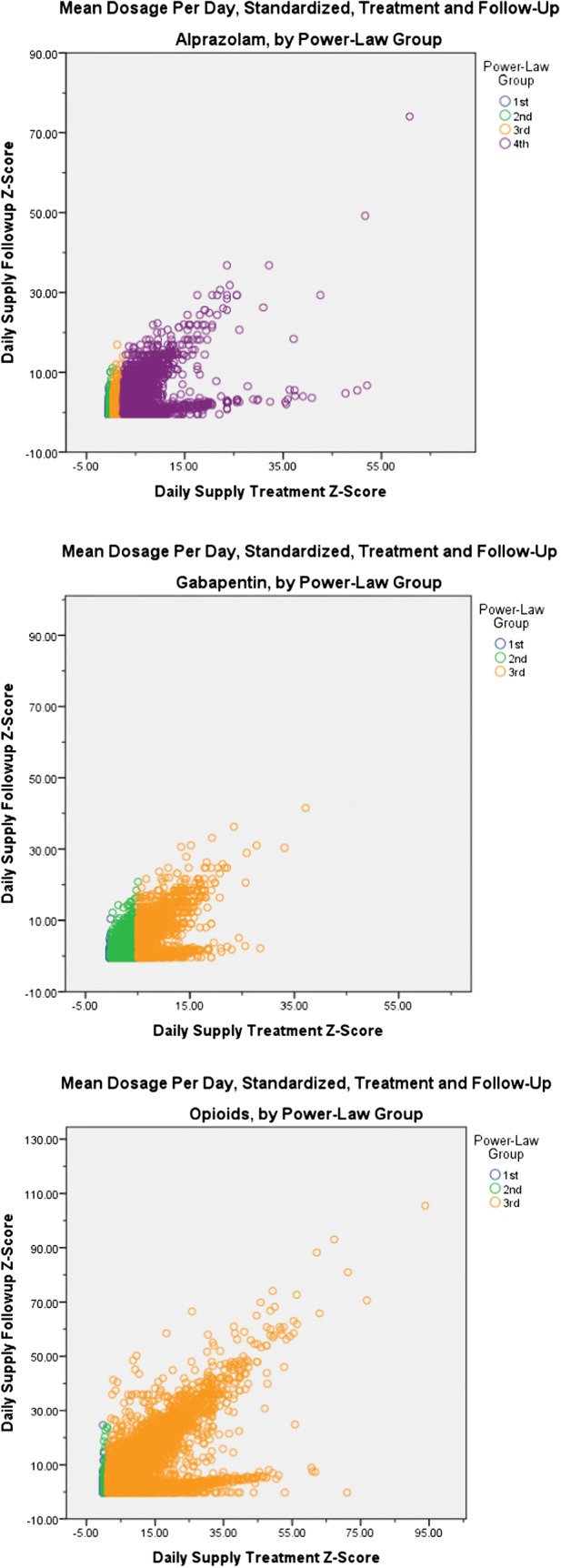
Criterion validity analyses: standardized mean dosage/day, baseline treatment and 6-month follow-up, by PLGs. *PLG* power-law group

From the 1-year baseline to 6-month follow-up periods for all 3 medications, mean daily dosages changed only modestly for most treated patients in PLG-1 and PLG-2 (Fig. 1). In contrast, for those in each top PLG, a distinctive splitting pattern occurred, in which a proportion of patients experienced substantial increases in dispensed dosage from baseline to follow-up. The LCA was dominated by Lorenz-25, with no visible distinctions among quartiles to characterize the threshold dosage at which the splitting pattern began (Additional file 1: Appendix S5).

For prediction of follow-up Lorenz-1 use of alprazolam and opioids, PLA had somewhat lower sensitivity compared with LCA (83.5–95.4% vs. 99.5–99.9%, respectively) but better specificity (98.2–98.8% vs. 75.5%) and much better PPV (34.5–45.3% vs. 4.0–4.6%; Table 2). Of those in the top PLG for alprazolam and opioids, respectively, 20.7 and 9.9% went from utilization < Lorenz-1 at baseline to Lorenz-1 in follow-up (i.e., incident Lorenz-1). For the same respective medications, only 2.5 and 1.3% in Lorenz-25 went on to incident Lorenz-1. For patients treated with gabapentin, PPV for incident Lorenz-1 was < 2% for both techniques.

**Table 2:** Criterion validity assessment: Lorenz-1 status and inpatient hospital use

Power law zone	Alprazolam (n = 463,203)				Gabapentin (n = 267,693)				Opioid (n = 2,077,393)			
	PLG1	PLG2	PLG3	PLG4	PLG1	PLG2	PLG3		PLG1	PLG2	PLG3	
N of cases	241,992	161,677	49,603	9931	174,989	90,590	2114		1,833,724	186,258	57,411	
Lorenz-1 in follow-up	0.0	0.0	1.6	45.3	0.0	1.4	58.5		0.0	0.5	34.5	
Lorenz-1 sensitivity, specificity, PPV, NPV^a^ (%)	83.5, 98.8, 45.3, 99.8				48.9, 99.7, 58.5, 99.5				95.4, 98.2, 34.5, 100.0			
Incident Lorenz-1 in follow-up	0.0	0.0	1.6	20.7	0.0	1.1	0.0		0.0	0.5	9.9	
Incident Lorenz-1 sensitivity, specificity, PPV, NPV^a^ (%)	69.9, 98.3, 20.7, 99.8				0.0, 99.2, 0.0, 99.6				85.5, 97.5, 9.9, 100.0			
IPH observation year	3.5	5.0	6.6	6.3	7.0	8.7	9.5		5.7	9.3	8.4	
IPH follow-up	3.6	5.0	6.6	7.3	6.7	8.4	9.4		4.5	8.1	8.2	
IPH % change	2.9	0.0	0.0	15.9	− 4.3	− 3.4	− 1.1		− 21.1	− 12.9	− 2.4	
IPH sensitivity, specificity, PPV, NPV (%)	3.5, 97.9, 7.3, 95.6				1.0, 99.2, 9.4, 92.7				4.6, 97.3, 8.2, 95.2			
SUD^b^ IPH observation year	0.6	1.2	2.0	2.0	1.4	1.7	2.6		0.7	2.3	2.6	
SUD^b^ IPH follow-up	0.6	1.2	2.2	2.8	1.3	1.6	2.5		0.7	2.2	2.9	
SUD^b^ IPH % change	0.0	0.0	10.0	40.0	− 7.1	− 5.9	− 3.8		0.0	− 4.3	11.5	
SUD IPH sensitivity, specificity, PPV, NPV^a^ (%)	5.7, 97.9, 2.8, 99.0				1.4, 99.2, 2.5, 98.6				9.4, 97.3, 2.9, 99.2			

Utilization quartile	Q1	Q2	Q3	Q4	Q1	Q2	Q3	Q4	Q1	Q2	Q3	Q4
N of cases	133,446	108,543	103,532	117,680	71,889	60,802	65,223	69,779	486,760	537,304	528,704	524,625
Lorenz-1 in follow-up	0.0	0.0	0.0	4.6	0.0	0.0	0.0	3.6	0.0	0.0	0.0	4.0
Lorenz-1 sensitivity, specificity, PPV, NPV^a^ (%)	99.5, 75.5, 4.6, 100.0				99.6, 74.6, 3.6, 100.0				99.9, 75.5, 4.0, 100.0			
Incident Lorenz-1 in follow-up	0.0	0.0	0.0	2.5	0.0	0.0	0.0	1.4	0.0	0.0	0.0	1.3
Incident Lorenz-1 sensitivity, specificity, PPV, NPV^a^ (%)	99.2, 75.1, 2.5, 100.0				99.1, 74.2, 1.4, 100.0				99.7, 75.0, 1.3, 100.0			
IPH observation year	3.2	4.0	4.7	6.0	6.4	7.1	8.2	8.9	2.8	4.7	7.3	9.3
IPH follow-up	3.4	3.9	4.7	6.1	6.1	6.9	7.7	8.5	3.2	3.8	5.2	7.4
IPH % change	6.2	− 2.5	0.0	1.7	− 4.7	− 2.8	− 6.1	− 4.5	14.3	− 19.1	− 28.8	− 20.4
IPH sensitivity, specificity, PPV, NPV^a^ (%)	34.6, 75.0, 6.1, 96.1				30.5, 74.3, 8.5, 93.1				38.0, 75.4, 7.4, 95.9			
SUD^b^ IPH observation year	0.5	0.8	1.1	1.7	1.2	1.4	1.5	1.7	0.3	0.5	0.8	1.8
SUD^b^ IPH follow-up	0.5	0.8	1.1	1.9	1.2	1.3	1.4	1.7	0.4	0.5	0.7	1.8
SUD^b^ IPH % change	0.0	0.0	0.0	11.8	0.0	− 7.1	− 6.7	0.0	33.3	0.0	− 12.5	0.0
SUD IPH sensitivity, specificity, PPV, NPV^a^ (%)	45.9, 74.8, 1.9, 99.2				31.6, 74.0, 1.7, 98.7				52.4, 75.0, 1.8, 99.4			

Earliest baseline year from January 1, 2013, through December 31, 2013, with follow-up from January 1, 2014, through June 30, 2014. Latest baseline year from July 1, 2014, through June 30, 2015, with follow-up from July 1, 2015, through December 31, 2015

*IPH* inpatient hospital, *NPV* negative predictive value, *PLG* power-law group, *PPV* positive predictive value, *Q* quartile, *SUD* substance use disorder

^a^Assuming that top PLG category and fourth quartile are predicted as at risk

^b^Diagnosis codes in Additional file 1: Appendix S4

For follow-up IPH, sensitivity was much less for PLA (1.0–4.6%) than for LCA (30.5–38.0%). However, both specificity and PPV were improved using PLA.

### Discussion

This proof-of-concept study applied PLA, which was originally developed to predict catastrophic events in physical systems, for the new purpose of predicting FWA development based solely on pharmacy claims data. For controlled substances, PLA performed better than did LCA at identifying a cohort of patients not currently engaging in, but at risk of developing, future FWA, with somewhat reduced sensitivity but better specificity and PPV. Notably, patients in the top PLGs, Lorenz-25, and Lorenz-1 were demographically and clinically similar at baseline in all respects other than pharmacy utilization. For a health care organization attempting to target FWA mitigation efforts to enrollees most in need of them, the improved PPV and specificity outcomes achieved with PLA are potentially important. However, in this preliminary analytic stage, PLA’s case-finding ability, especially for IPH, was not sufficient for widespread application. Several areas for future development are indicated.

Foremost, results suggest that patients in top PLGs may be at increased risk of future abuse/diversion; yet, top-PLG status is clearly not a perfect predictor of FWA. Future analyses should assess FWA predictors in subsamples limited to top-PLG patients. Bollinger-band analysis, which has been used to predict surges in use of intensive-care services [19], might provide additional insights into medication demand changes in top PLGs. Perhaps the lower PPVs for IPH compared with Lorenz-1 indicate detection of diversion, rather than consumption, of medication.

Additionally, the most common use of PLA in the physical sciences is performed at the regional level, predicting federal expenditures on catastrophic events for specific geographic areas [12]. The increasing availability of data from prescription drug monitoring programs (PDMP) [20] may represent an opportunity to apply PLA to regional utilization and mortality data, thereby providing health care payers with better information about which regions are most at risk, and which PDMP features may most effectively mitigate FWA-related harms.

### Conclusion

Application of PLA to pharmacy claims data is a promising new method for identifying patients at risk of FWA but needs additional refinement prior to widespread use. Potential future applications may include proactive management of known or emerging medications of abuse [21].

## Limitations

If PLA predicts fundamental system change, it is not clear why only a portion of patients in each top PLG went on to experience large dosage increases, indicating potential FWA. Our application of PLA to *all* treated patients with ≥ 2 claims may have been overly broad—perhaps analogous to measuring a catastrophic event of *any* type, rather than the technique’s original purpose of measuring only *one* type of catastrophic event. Future applications might limit samples to more restricted subgroups and outcomes specific to key clinical situations; for example, development of FWA in patients with SUD, or of SUD in patients treated with opioids for chronic noncancer pain [22]. Similarly, since recent work has suggested that gabapentin misuse is likely only in patients who either have a SUD diagnosis or are concomitantly using opioids [23], a future sample might be limited to patients with concomitant gabapentin/opioid use to improve PLA’s PPV for gabapentin misuse. Moreover, the 6-month follow-up period, the maximum possible for this dataset and design, was not long enough to measure long-term developments. Additional analyses following patients for longer time periods may yield more information about FWA development.

## Additional file

**Additional file 1: Appendix S1.** Power-law file preparation, alprazolam, baseline treatment year. Example calculation to illustrate how aggregated data are prepared for power-law analysis. *CF* cumulative frequency, *mg* milligrams. 540,752 = total n of alprazolam-treated patients meeting sample criteria. **Appendix S2.** Power-Law Curves. Excel graphics showing (a) logarithmically transformed 2-dimensional plots of cumulative frequency against event magnitude and (b) transition points for each power-law zone. **Appendix S3.** Linear Equations by Power-Law Zones and Medication. Slopes and R^2^ for each power-law zone. **Appendix S4.** Diagnoses. International classification of diseases, diagnosis-related group, place of service, and current procedural terminology codes for all diagnoses measured in the study. ^a^ Measured using both ICD-9 and ICD-10 coding because follow-up period included dates of service on and after October 1, 2015. *CPT* current procedural terminology, *HCPCS* healthcare common procedure coding system, *ICD* international classification of diseases. **Appendix S5.** Criterion validity analyses: standardized mean dosage/day, baseline treatment and 6-month follow-up, by utilization quartiles.

## Abbreviations

US: United States
FWA: fraud, waste, or abuse
LCA: Lorenz-curve analysis
mg: milligrams
MME: morphine-milligram equivalents
IPH: inpatient hospitalization
PLA: power-law analysis
PLZ: power-law zones
PLG: power-law groups
PPV: positive predictive value
SUD: substance use disorder
PDMP: prescription drug monitoring program
CF: cumulative frequency
Q: quartile
CPT: current procedural terminology
HCPCS: Healthcare Common Procedure Coding System
ICD: International Classification of Diseases
