# Supplementary material for: Use of power-law analysis to predict abuse or diversion of prescribed medications: proof-of-concept mathematical exploration
Source: BMC Res Notes. 2018 Jul 31;11:523. doi: 10.1186/s13104-018-3632-y (PMC6069871; doi:10.1186/s13104-018-3632-y)

Appendix S1. Power-Law File Preparation, Alprazolam, Baseline Treatment Year

Description	N of Patients	Total Mg Dispensed	Magnitude (Mg/Patient)	Log_{10} of Magnitude	CF	Log_{10} of CF
Top 0.1% (99.9th %ile)	541	5,025,987	9290.2	3.968	541	2.733
Next 0.1%	541	3,026,105	5593.5	3.748	1082	3.034
Next 0.1%	541	2,572,667	4755.4	3.677	1623	3.210
Calculation proceeded through each band, finishing with:						
Bottom 1%	5407	16,946	3.1	0.496	540,752	5.733

Appendix S2. Power-Law Curves

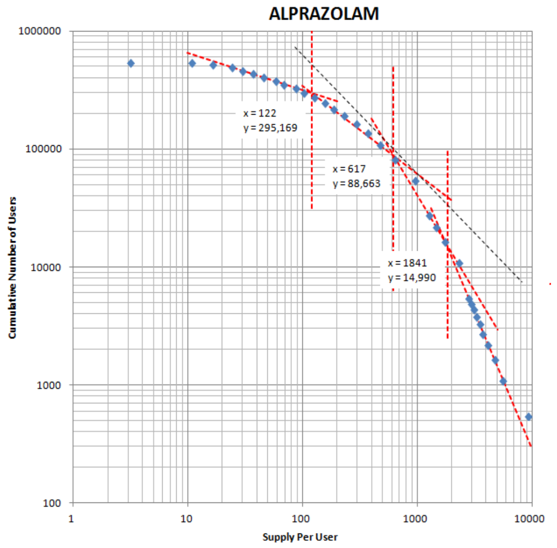

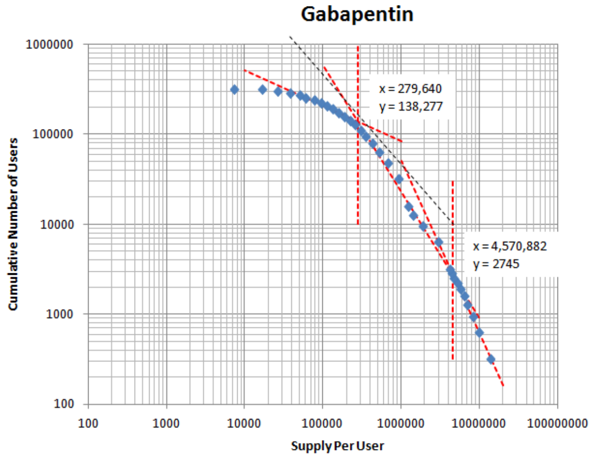

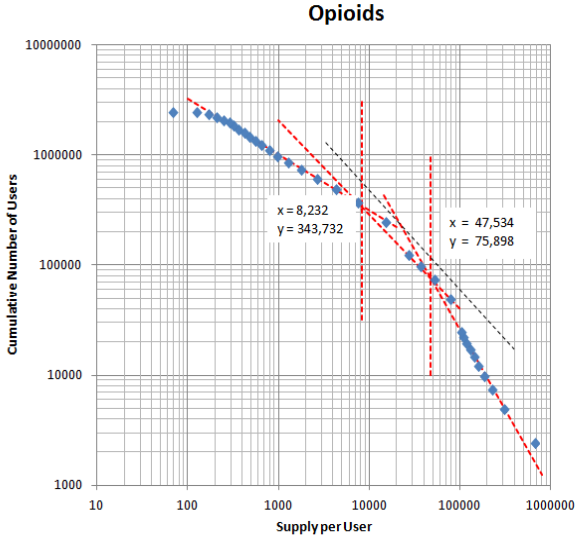

Appendix S3. Linear Equations by Power-Law Zones and Medication

	R^2	Slope (b)	R^2	Slope (b)	R^2	Slope (b)	R^2	Slope (b)
Alprazolam	0.998254	-2.3446	0.998761	-1.53297	0.990357	-0.74078	0.99377	-0.31594
Gabapentin	0.993926	-1.92284	0.984165	-1.40283	0.972815	-0.39091	N/A	N/A
Opioids	0.99792	-1.48948	0.960612	-0.85083	0.998227	-0.50942	N/A	N/A

Appendix S4. Diagnoses

Diagnosis	Codes
Anxiety	**ICD-9 Code:** 300 Anxiety, dissociative and somatoform disorders
Cancer<br>DRG 146-148 ENT malignancy<br>374-376 digestive malignancy<br>435-437 hepatobiliary malignancy<br>582-583 mastectomy for malignancy<br>597-599 malignant breast disorders<br>715-716 male reproductive system procedures for malignancy<br>722-724 male reproductive system malignancy<br>737-741 procedure for ovarian/adnexal malignancy<br>754-756 female reproductive system malignancy<br>820-825 lymphoma and leukemia<br>834-839 leukemia<br>846-848 chemotherapy with leukemia	**ICD-9 Codes**<br>140-209 Malignant neoplasms<br>338.3 Neoplasm-related pain<br>V58.1 Encounter for antineoplastic chemotherapy and immunotherapy
Chronic kidney disease (moderate to severe)<br>DRG 682-684 renal failure or 685 admit for renal dialysis or revenue code 800-809 or 822-839 or 880-889 (dialysis) or diagnosis code	**ICD-9 Codes**<br>585.3 Chronic kidney disease, Stage III (moderate)<br>585.4 Chronic kidney disease, Stage IV (severe)<br>585.5 Chronic kidney disease, Stage V; 585.6 End stage renal disease
Insomnia	**ICD-9 Code:** 780.5 Sleep disturbances
Pain, chronic, musculoskeletal, or neuropathic	**ICD-9 Codes**<br>338.2x Chronic pain<br>338.4x Chronic pain syndrome<br>707 Chronic ulcer of skin<br>780.96 Generalized pain<br>V58.64 Long-term (current) use of non-steroidal anti-inflammatories (NSAID)<br>V66.7 Encounter for palliative care<br>710.xx-719.xx Arthropathies And Related Disorders<br>720.xx-724.xx Dorsopathies<br>725.xx-729.xx Rheumatism, Excluding The Back<br>730.xx-739.xx Osteopathies, Chondropathies, And Acquired Musculoskeletal Deformities<br>250.6x Diabetes with neurological manifestations<br>353 Nerve root and plexus disorders<br>354 Mononeuritis of upper limb and mononeuritis multiplex<br>355 Mononeuritis of lower limb and unspecified site<br>356 Hereditary and idiopathic peripheral neuropathy<br>357 Inflammatory and toxic neuropathy<br>357.2 Polyneuropathy in diabetes
**Substance Use Disorder** [a]<br>ICD codes shown to the right<br>**OR** DRG=894-897 (alcohol/drug abuse or dependence)<br>**OR** place of service=55 or 57(substance abuse facility) **OR** service category=31110-31769 (substance abuse services) **OR** revenue code=116, 126, 136, 146, or 156 (detox bed) or 1002 (chemical dependency) **OR** HCPCS:<br>H0005 Alcohol and/or drug services; group counseling by a clinician<br>H0006 Alcohol and/or drug services; case management<br>H0007 Alcohol and/or drug services; crisis intervention (outpatient)<br>H0008 Alcohol and/or drug services; sub-acute detoxification (hospital inpatient)<br>H0009 Alcohol and/or drug services; acute detoxification (hospital inpatient)<br>H0010 Alcohol and/or drug services; sub-acute detoxification (residential)<br>H0011 Alcohol and/or drug services; acute detoxification (residential).<br>H0012 Alcohol and/or drug services; sub-acute detoxification (residential)<br>H0013 Alcohol and/or drug services; acute detoxification (residential)<br>H0014 Alcohol and/or drug services; ambulatory detoxification	**ICD-9 Codes**<br>291 Alcohol-induced mental disorders<br>292 Drug-induced mental disorders<br>303.xx Alcohol dependence syndrome<br>304.xx Drug dependence<br>305.xx Nondependent abuse of drugs<br>965.0 Poisoning by opiates and related narcotics<br>967 Poisoning by sedatives and hypnotics<br>969.4 Poisoning by benzodiazepine-based tranquilizers<br>970 Poisoning by central nervous system stimulants<br>E851 Accidental poisoning by barbiturates<br>E852 Accidental poisoning by other sedatives and hypnotics<br>E853 Accidental poisoning by tranquilizers<br>E854.1 Accidental poisoning by psychodysleptics [hallucinogens]<br>E854.2 Accidental poisoning by psychostimulants<br>E854.3 Accidental poisoning by central nervous system stimulants<br>E850.0 Accidental poisoning by heroin<br>E850.1 Accidental poisoning by methadone<br>E850.2 Accidental poisoning by other opiates and related narcotics

H0015 Alcohol and/or drug services; intensive outpatient (treatment program)<br>H0016 Alcohol and/or drug services; medical/somatic<br>H0047 Alcohol and/or other drug abuse services, not otherwise specified<br>H2034 Alcohol and/or drug abuse halfway house services, per diem<br>H2035 Alcohol and/or other drug treatment program, per hour<br>H2036 Alcohol and/or other drug treatment program, per diem<br>G0396 Alcohol and/or substance (other than tobacco) abuse structured assessment and intervention<br>G0397 Alcohol and/or substance (other than tobacco) abuse structured assessment and intervention<br>**OR** CPT codes 99408 or 99409 (SUD assessment and intervention)	**ICD-10 Codes**<br><br>F10 Alcohol related disorders<br>F11 Opioid related disorders<br>F12 Cannabis related disorders<br>F13 Sedative, hypnotic, or anxiolytic related disorders<br>F14 Cocaine related disorders<br>F15 Other stimulant related disorders<br>F16 Hallucinogen related disorders<br>F18 Inhalant related disorders<br>F19 Other psychoactive substance related disorders<br>T40 Poisoning by, adverse effect of and underdosing of narcotics and psychodysleptics excluding codes indicting underdosing

Appendix S5. Criterion Validity Analyses: Standardized Mean Dosage/Day, Baseline Treatment and 6-Month Follow-Up, by Utilization Quartiles

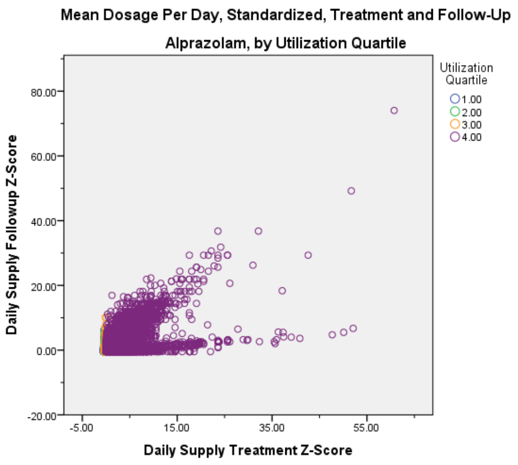

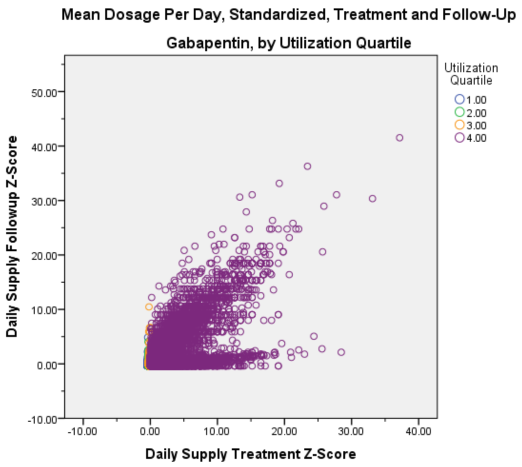

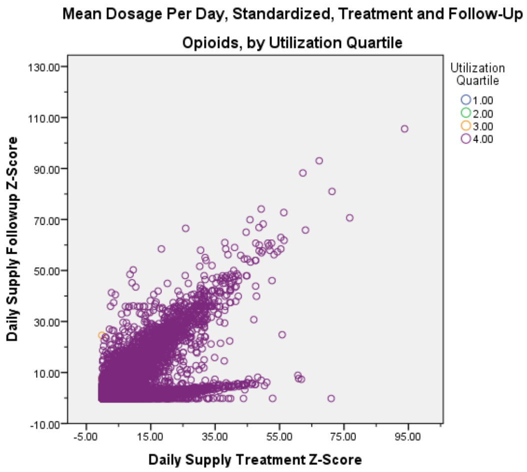

Supplement: Supplementary file 1 — Additional file 1: Appendix S1. Power-law file preparation, alprazolam, baseline treatment year. Example calculation to illustrate how aggregated data are prepared for power-law analysis. CF cumulative frequency, mg milligrams. 540,752 = total n of alprazolam-treated patients meeting sample criteria. Appendix S2. Power-Law Curves. Excel graphics showing (a) logarithmically transformed 2-dimensional plots of cumulative frequency against event magnitude and (b) transition points for each power-law zone. Appendix S3. Linear Equations by Power-Law Zones and Medication. Slopes and R2 for each power-law zone. Appendix S4. Diagnoses. International classification of diseases, diagnosis-related group, place of service, and current procedural terminology codes for all diagnoses measured in the study. a Measured using both ICD-9 and ICD-10 coding because follow-up period included dates of service on and after October 1, 2015. CPT current procedural terminology, HCPCS healthcare common procedure coding system, ICD international classification of diseases. Appendix S5. Criterion validity analyses: standardized mean dosage/day, baseline treatment and 6-month follow-up, by utilization quartiles. [file 13104_2018_3632_MOESM1_ESM.pdf]
